# A Synthetic Ecology Perspective: How Well Does Behavior of Model Organisms in the Laboratory Predict Microbial Activities in Natural Habitats?

**DOI:** 10.3389/fmicb.2016.00946

**Published:** 2016-06-15

**Authors:** Zheng Yu, Sascha M. B. Krause, David A. C. Beck, Ludmila Chistoserdova

**Affiliations:** ^1^Department of Chemical Engineering, University of Washington, SeattleWA, USA; ^2^Department of Microbiology, University of Washington, SeattleWA, USA; ^3^eScience Institute, University of Washington, SeattleWA, USA

**Keywords:** synthetic community, methylotrophy, methanotroph, *Methylobacter*, *Methylosarcina*, *Methylomonas*, Lake Washington

## Abstract

In this perspective article, we question how well model organisms, the ones that are easy to cultivate in the laboratory and that show robust growth and biomass accumulation, reflect the dynamics and interactions of microbial communities observed in nature. Today’s -omics toolbox allows assessing the genomic potential of microbes in natural environments in a high-throughput fashion and at a strain-level resolution. However, understanding of the details of microbial activities and of the mechanistic bases of community function still requires experimental validation in simplified and fully controlled systems such as synthetic communities. We have studied methane utilization in Lake Washington sediment for a few decades and have identified a number of species genetically equipped for this activity. We have also identified co-occurring satellite species that appear to form functional communities together with the methanotrophs. Here, we compare experimental findings from manipulation of natural communities involved in metabolism of methane in this niche with findings from manipulation of synthetic communities assembled in the laboratory of species originating from the same study site, from very simple (two-species) to rather complex (50-species) synthetic communities. We observe some common trends in community dynamics between the two types of communities, toward representation of specific functional guilds. However, we also identify strong discrepancies between the dominant methane oxidizers in synthetic communities compared to natural communities, under similar incubation conditions. These findings highlight the challenges that exist in using the synthetic community approach to modeling dynamics and species interactions in natural communities.

## From Model Organisms to Model Communities?

For centuries, Biology has relied on model organisms for understanding the details of metabolism, regulation, evolution trends, etc. For example, rats, mice, nematode worms and yeast are all used as models for understanding complex metabolism and regulation in humans (Persico et al., 2005; Simmons, 2008). Microbiology has also relied on model organisms, for over a hundred years, the models typically selected for their ease of cultivation and laboratory manipulation (Bennett and Hughes, 2009). While early studies in microbiology were mainly focused on human pathogens, the importance of microbes in the environment has been eventually acknowledged, and interest in environmental microbes has lead through observing a great plating anomaly (Staley and Konopka, 1985), to uncovering the fact that most microbes have remained unknown because they remained uncultivated (Puspita et al., 2012). As we are now well aware of this fact, and also of the fact that laboratory media designed for human pathogens are not necessarily appropriate for cultivating environmental microbes (Janssen et al., 2002; Joseph et al., 2003; Henson et al., 2016), we are in a position of being able to select model organisms not purely based on the ease of cultivation, but based on their relevance and importance for a specific environmental activity. A synthetic community approach presents an attractive alternative to studying natural communities, through reduced complexity and through full experimental control (Jessup et al., 2004; Großkopf and Soyer, 2014). However, with multiple choices of models, several important questions need to be considered. How does one choose which model to employ in laboratory simulations? How well does manipulation in the laboratory reflect behavior in natural environments? Even more importantly, how do observations from single-species behavior reflect their behavior as parts of microbial assemblages involved in specific environmental activities?

Methane oxidation is an important environmental process that mitigates release of large quantities of methane, mostly generated biogenically, via degradation of complex organic matter (Singh et al., 2010). This process is carried out by special microbes that differ in their nature, dependent on a specific environment. In anoxic marine environments, these are mainly anaerobic methanotrophic archaea (ANME) that use a reverse methanogenesis pathway to oxidize methane, linking it to reduction of sulfate (Knittel and Boetius, 2009; Ruff et al., 2015), nitrate (McGlynn et al., 2015; Wegener et al., 2015), or to insoluble metal reduction (Beal et al., 2009). In terrestrial habitats, including extreme environments, the dominant types are bacteria belonging to three distinct phyla: Proteobacteria, Verrucomicrobia, and candidate phylum NC10, which activate methane with oxygen and use specialized pathways for carbon assimilation (Chistoserdova, 2011, 2015). So far, only representatives of Proteobacteria and Verrucomicrobia have been isolated in pure cultures (Chistoserdova and Lidstrom, 2013).

Lake Washington is a prototypical freshwater lake where active methane production takes place in the anoxic layers of the sediment and where most of the methane produced is consumed in the upper layer of the sediment, which is characterized by steep counter-gradients of methane and oxygen (Kuivila et al., 1988; Auman et al., 2000). We have studied the communities involved in methane oxidation in this niche for decades, using pure cultures of methylotrophs as well as culture-independent approaches (Chistoserdova et al., 2013; Chistoserdova, 2015). Recent findings suggest that complex, interacting functional communities, rather than methanotroph species alone are involved in methane oxidation in this environment (Beck et al., 2013; Chistoserdova, 2015). However, the data from different approaches have not always agreed with each other. The species that are easy to cultivate and that demonstrate robust growth in the laboratory mainly belong to the genera *Methylomonas* (gammaproteobacteria) and *Methylosinus* (alphaproteobacteria; Auman et al., 2000). In contrast, DNA stable isotope probing (DNA-SIP) with ^13^C-methane suggested that *Methylobacter* species might be the dominant active type in laboratory setups approximating natural conditions (*in situ* temperature, natural lake water; Kalyuzhnaya et al., 2008; Beck et al., 2013). It took significant efforts to isolate *Methylobacter* strains from Lake Washington sediment in pure cultures (Chistoserdova, 2015; Oshkin et al., 2015), and, in terms of laboratory ‘fitness’ they underperform as compared to the *Methylomonas* or *Methylosinus* strains, revealing slower growth under standard conditions, slow colony formation and also a psychrophilic character (Kalyuzhnaya et al., 2015 and unpublished). These discrepancies between laboratory ‘fitness’ and *in situ* activity were further addressed through microcosm manipulation of native sediment samples, under conditions of varying oxygen partial pressures. In these experiments, both *Methylomonas* and *Methylosinus* types were consistently outperformed by the *Methylobacter* types, under low oxygen partial pressures, or by *Methylosarcina* types, under high oxygen partial pressures (Hernandez et al., 2015; Oshkin et al., 2015). The non-methanotroph species that were detected at high frequencies in these communities were non-methane utilizing methylotrophs of the *Methylophilaceae* family and some non-methylotrophic heterotrophs, most prominently *Flavobacteriales* and *Burkholderiales* (Hernandez et al., 2015; Oshkin et al., 2015).

Our long-term goal is to understand how natural communities active in methane oxidation perform in natural environments and what the main factors are that control their activities. As part of this goal, we further addressed discrepancies that might exist between laboratory performances (‘fitness’) of major players, as observed at pure culture level, compared to activities/conditions approximating environmental settings. The existence of such discrepancies would have major implications for the selection of laboratory models and the experimental conditions. It would affect not only how we interpret the data on their performance, but also how we scale up these findings to the level of predicting and modeling global elemental cycles. Ultimately, we question whether our model choices and their manipulation in simplified laboratory settings allow for realistic interpretation of microbial activities in natural environmental settings.

## Synthetic Community Dynamics Do Not Exactly Follow Natural Community Dynamics

**FIGURE 1:**
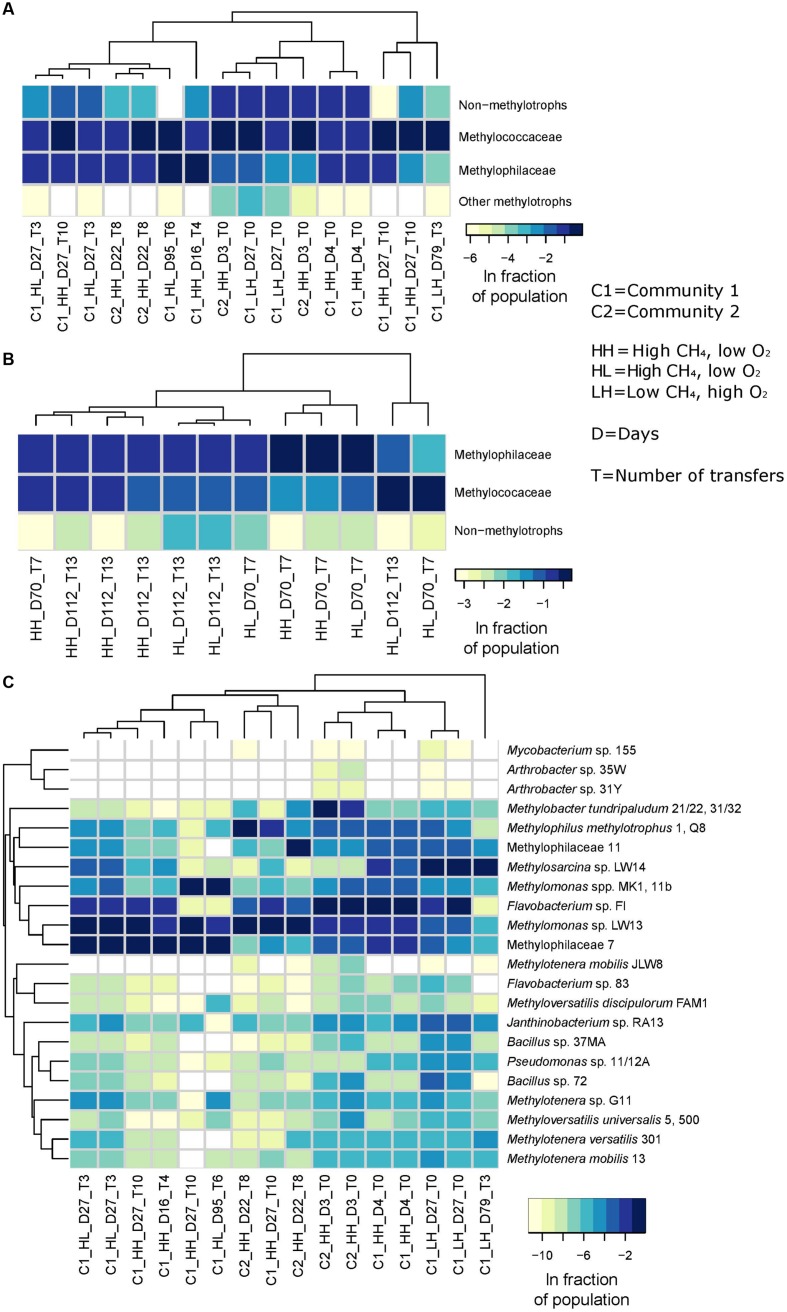
**Species dynamics in synthetic communities resemble dynamics of natural communities at the level of specific functional guilds but not at the species level.** Two communities (C1 and C2) were assembled of 50 strains, all previously isolated from Lake Washington sediment. The methanotrophs: (*Methylomonas* sp. LW13, *Methylomonas* sp. MK1, *Methylomonas* sp. 11b, *Methylobacter tundripaludum* 21/22, *Methylobacter tundripaludum* 31/32, *Methylosarcina lacus* LW14 (Kalyuzhnaya et al., 2015), *Methylosinus* sp. PW1, *Methylosinus* sp. LW3, *Methylosinus* sp. LW4, *Methylosinus* sp. LW5 (Beck et al., 2015); the *Methylophilaceae*: *Methylotenera mobilis* JLW8, *Methylotenera versatilis* 301, *Methylovorus glucosotrophus* SIP3-4 (Lapidus et al., 2011), *Methylotenera mobilis* 13, *Methylotenera* sp. G11, *Methylophilaceae* 7, *Methylophilaceae* 73s, *Methylophilaceae* 11, *Methylophilus methylotrophus* 1, *Methylophilus methylotrophus* 5, *Methylophilus methylotrophus* Q8 (Beck et al., 2014; McTaggart et al., 2015b); non-*Methylophilaceae* methylotrophs: *Arthrobacter* sp. 31Y, *Arthrobacter* sp. 35W, *Arthrobacter* sp. MA-N2, *Bacillus* sp. 37MA, *Bacillus* sp. 72, *Mycobacterium* sp. 141, *Mycobacterium* sp. 155 (McTaggart et al., 2015a), *Aminobacter* sp. 108, *Ancylobacter* sp. 117, *Ancylobacter* sp. 202, *Ancylobacter* sp. 501b, *Hyphomicrobium* sp. 99, *Hyphomicrobium* sp. 802, *Labrys methylaminiphilus* JLW10, *Methylobacterium* sp. 10, *Methylobacterium* sp. 77, *Methylobacterium* sp. 88A, *Methylopila* sp. 73B, *Methylopila* sp. 107, *Paracoccus* sp. N5, *Xanthobacter* sp. 91, *Xanthobacter* sp. 126 (Beck et al., 2015), *Methyloversatilis discipulorum* FAM1, *Methyloversatilis universalis* FAM5, *Methyloversatilis universalis* FAM500 (Smalley et al., 2015), non-methylotrophic heterotrophs: *Pseudomonas* sp. 11/12A, *Janthinobacterium* sp. RA13, *Flavobacterium* sp. 83, *Flavobacterium* sp. Fl (McTaggart et al., 2015c,d,e). In C1, the 50 strains were mixed in equal proportions based on optical density (OD_600_), and in C2, they were mixed based on cell counts as determined by flow cytometry. The main difference between C1 and C2 was in the proportion of the methanotrophs and the *Bacillus* strains whose cells have larger size, thus they were less abundant in C1 compared to C2. Incubations were carried out at 18°C in vials, as previously described (Hernandez et al., 2015; Oshkin et al., 2015). Initial OD_600_ was 0.1. Gas phase was replenished daily. When cultures reached OD_600_ 0.5, they were transferred with 10-fold dilution into fresh medium. 16S rRNA gene fragment (iTag) sequencing and analysis were carried out as previously described (Hernandez et al., 2015; Oshkin et al., 2015), except for the clustering was done at 98% sequence identity cutoff (data archived with Bioproject Number Pending). **(A)** Relative abundances of *Methylococcaceae, Methylophilaceae*, other methylotrophs, and non-methylotrophs in synthetic communities. **(B)** Relative abundances of *Methylococcaceae, Methylophilaceae*, and non-methylotrophs in natural communities, drawn from a partial dataset from Hernandez et al. (2015). **(C)** Relative abundances of individual strains in synthetic communities.)

We assembled synthetic communities of 10 methanotrophs, 36 non-methanotrophic methylotrophs, and 4 non-methylotrophic heterotrophs (details in **Figure 1**), to address the discrepancies between the performance of natural communities in semi-*in situ* conditions and laboratory performance of pure culture isolates. Even though all 50 organisms originated from Lake Washington sediment, we did not follow any specific design for community structure, assuming that species that thrive in the specific incubation conditions that we apply would increase in their abundance and species that are not fit for these conditions would decrease in their abundance. Live cultures were mixed together in similar cell counts and were incubated for several weeks, with periodic transfers and dilutions, as previously described (Hernandez et al., 2015; Oshkin et al., 2015), under three different gas phase conditions: high methane/low oxygen (HL, methane/air in headspace 25/5% (V/V), the remainder balanced with N_2_), low methane/high oxygen (LH, methane/air in headspace 0.5/75%, the remainder balanced with N_2_), and high methane/high oxygen (HH, methane/air in headspace 25/75%; more details in **Figure 1**). While the first two conditions mimicked gas concentrations in the opposing parts of methane and oxygen gradients in the natural lake sediment, the third condition represented a typical laboratory setting. We addressed two main questions: (i) whether *Methylomonas* and *Methylosinus* would outperform *Methylobacter* and *Methylosarcina*, to reflect their robust performance in the laboratory, and (ii) whether species other than *Methylophilaceae, Flavobacteriales* or *Burkholderiales* would persist in methane-fed communities, also based on laboratory performance of the cultivated species. In each of the communities, rapid loss of species complexity was observed after a few days of incubation, as previously observed for natural communities of high complexity (Hernandez et al., 2015; Oshkin et al., 2015). Similarly to the natural communities, methanotrophs of the family *Methylococcaceae* and non-methanotrophic methylotrophs of the family *Methylophilaceae* were found at relatively high abundances (**Figures 1A,B**) while methanotrophs of the family *Methylocystaceae* were undetectable. However, significant differences compared to natural community dynamics were uncovered in terms of the dominant methanotrophs. While *Methylobacter* species were found as dominant under the HL condition in natural samples (Hernandez et al., 2015), they were only detected at relatively high abundances in some early HL samples, quickly outcompeted by the *Methylomonas* species over time (**Figure 1C**). The dynamics of the *Methylosarcina* population also differed from the ones previously observed in natural communities. While *Methylosarcina* was reported to outperform *Methylobacter* under the HH condition (Hernandez et al., 2015), in synthetic communities *Methylosarcina* was outcompeted by *Methylomonas* under both the HH and HL conditions, while *Methylosarcina* outcompeted both *Methylobacter* and *Methylomonas* under the LH condition (**Figure 1C**).

## Three Laboratory Models Demonstrate Differential Fitness Dependent on Community Complexity

**FIGURE 2:**
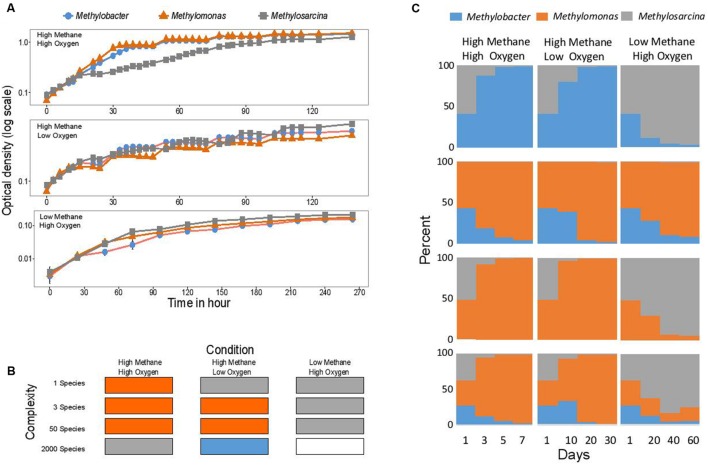
**Fitness/performance of three model methanotrophs in pure cultures and in communities show discrepancies.** **(A)** Growth curves measured for pure cultures of *Methylomonas* sp. LW13, *M. tundripaludum* 21/22 and *M. lacus* LW14, in 30 ml tubes filled with 15 ml liquid cultures, in triplicates (standard errors shown). Tubes were incubated with shaking at 18°C, in angled racks. Gas composition was replenished daily. **(B)** Relative abundance of each methanotroph in mixed cultures, over time, as measured by quantitative polymerase chain reaction (qPCR), essentially as described in Chu and Lidstrom (2016). Cultures were incubated in vials with transfers/dilutions, as above. Number of copies of the *orfY* gene (unknown function in formaldehyde oxidation, Kalyuzhnaya et al., 2005) were measured in each case (gene 03197 in *Methylomonas* sp. LW13, gene 3399 in *M. tundripaludum* 21/22 and gene 3547 in *M. lacus* LW14), using primer pairs specific to each organism. Genome data and annotations are available at https://img.jgi.doe.gov. At least three biological replicates were tested, with average standard error <10% (not shown). **(C)** Summary of the strains with highest performance in each experiment. Data for natural communities (at least 2000 species) are from Hernandez et al., 2015. Blank, no data.

We next measured growth curves for pure cultures of three methanotroph species, *Methylomonas, Methylobacter*, and *Methylosarcina*. We also prepared simple two- and three-species mixtures, applying the same incubation conditions as described above (**Figures 2A,B**). The results from these incubations clearly demonstrate that the performance/fitness of the three model organisms, as pure cultures, did not correlate well with their performance/fitness as parts of synthetic communities, even when simple two- to three-species communities were considered (**Figure 2C**). In turn, their performance/fitness as parts of synthetic communities did not correlate well with the performance/fitness of their closely related counterparts as parts of natural communities (Hernandez et al., 2015 and unpublished; Oshkin et al., 2015; **Figure 2C**). However, performance/fitness of the simple (3-species) and the complex (50 species) synthetic communities showed some agreement (**Figure 2C**). Thus, we conclude that synthetic communities, at least as applied to methane-oxidizing communities and in the conditions used, appear to behave differently from the natural communities. These observations question, more generally, our reliance on data from laboratory manipulations for global predictions as related to microbe-mediated environmental processes.

## Path Toward Realistic Synthetic Communities?

Microbial communities involved in methane metabolism were used here as one example of a functional microbial assemblage involved into a specific biogeochemical process. In microbial ecology, and in ecology in general, fitness and performance of individual species are often considered as predictors of success in natural communities. However, the necessity of measuring performance in natural settings has been advocated (Irschick, 2003). We here present the discrepancies between fitness/performance of model methanotroph species in laboratory simulations, compared to fitness/performance of their closely related counterparts in natural communities. We intentionally tested semi-randomly assembled synthetic communities, mixing together 50 strains that have been previously isolated from Lake Washington sediment, most of them methylotrophs, with expectation that these synthetic communities would behave similarly to the natural communities, selecting for a smaller subset of active species under each specific gas phase condition. Indeed, we observed rapid loss of the species not previously seen in natural communities active in methane oxidation (Kalyuzhnaya et al., 2008; Beck et al., 2013; Hernandez et al., 2015; Oshkin et al., 2015), in contrast to the published data on enhanced performance of randomly assembled species in methane-oxidizing activity (Stock et al., 2013; Ho et al., 2014). We further observed that species compositions of the synthetic communities, when assessed at the family/order level, resembled those of natural communities, indicating a certain consistency at higher phylogenetic levels. However, at the genus level, the methane-oxidizing species behaved differently in synthetic communities compared to natural communities, with the *Methylomonas* spp., hardly seen in natural communities, showing robust performance, in agreement with their documented growth characteristics (Soni et al., 1998; Hoefman et al., 2012). In fact, none of our methanotroph models demonstrated behavior that correlated with their behavior in the natural sediment, assessed through DNA-SIP experiments or through microcosm manipulations. We can exclude inactive states of the *Methylomonas* and *Methylosinus* types in natural samples as we were able to detect their respective rRNA and mRNA molecules in the native sediment (Nercessian et al., 2005), and we can enrich them in certain conditions, such as elevated temperatures (30°C), at which *Methylobacter* species are inactive (Auman et al., 2000 and unpublished). Strikingly, *Methylosarcina* species, shown to outperform *Methylobacter* in the conditions of high oxygen in natural communities underperformed both *Methylobacter* and *Methylomonas* in synthetic communities under these conditions. There are two likely explanations for these discrepancies. (i) The strains that we successfully isolated in pure cultures poorly reflect strains that are active in the natural habitat. However, we do have evidence of their close relatedness at the genomic level (Oshkin et al., 2015 and unpublished). (ii) More likely, these discrepancies are due to the complexity of the natural communities that was not recaptured with either simple (2/3 strain) or more complex (50 strain) synthetic community models. In natural communities, other functional guilds may have major roles in shaping the community structure, including species not involved in primary methane utilization or co-metabolism. These may be both synergistic and antagonistic, for example, predatory species, species harboring predatory plasmids or phages, or free-living phages. We conclude that, overall, there are tradeoffs between manipulation of natural communities and synthetic communities consisting of convenient models, allowing for strict experimental control. Ultimately, while some fundamental questions could be addressed through manipulation of very simple synthetic communities (Großkopf and Soyer, 2014; Ponomarova and Patil, 2015), understanding the finer details of interspecies interactions might require experiments with communities reflecting more precisely activities of natural communities. We propose that synthetic communities should be modeled based on observations from natural communities, under conditions approximating natural conditions as much as reasonably possible in laboratory settings. Thus, an intelligent community design should be applied instead of random selection of species. Such synthetic communities would provide a more realistic representation of a natural process, in the laboratory. One then can use their signatures such as transcripts, proteins and metabolites to gain a more realistic understanding of a natural process. Specific cross-talk mechanisms between the community partners in such communities can also be tested via knock out mutant manipulation.

## Author Contributions

LC and ZY designed the experiments, ZY carried out experiments, SK carried out flow cytometric counting, DB carried out iTag data clustering. ZY and DB prepared figures. LC, ZY, SK, and DB wrote the manuscript.

## Conflict of Interest Statement

The authors declare that the research was conducted in the absence of any commercial or financial relationships that could be construed as a potential conflict of interest.
